# The Supplementation of Cinnamaldehyde in Semen Extender to Improve the Post‐Thaw Sperm Quality of Kajli Ram Semen

**DOI:** 10.1002/vms3.70484

**Published:** 2025-07-18

**Authors:** Saima Qadeer, Asma Ashraf, Naila Perveen, Muhammad Asad, Asma Ul Husna, Asima Azam, Muhammad Umer Farooq, Abdullah S. Alhomida, Haseeb A. Khan

**Affiliations:** ^1^ Department of Zoology Division of Science and Technology University of Education Lahore Pakistan; ^2^ Department of Biology University of Haripur Haripur Pakistan; ^3^ Department of Zoology Shaheed Benazir Bhutto Women University Peshawar Pakistan; ^4^ Department of Biochemistry College of Science King Saud University Riyadh Saudi Arabia

**Keywords:** antioxidant, cinnamaldehyde, cryopreservation, Kajli ram, semen

## Abstract

Cinnamaldehyde, a component of cinnamon bark extract, acts as a potential antioxidant by enhancing the activities of catalase, superoxide dismutase and glutathione peroxidase in various cell and animal models. This study aimed to evaluate the protective effects of cinnamaldehyde on the quality of Kajli ram sperm at 4°C and after freezing. Semen samples were obtained from 12 Kajli rams through an artificial vagina maintained at 42°C. Each ejaculate was divided into five aliquots and extended in a tris‐citric acid extender with different concentrations of cinnamaldehyde (0%, 0.5%, 1.0%, 2.5% and 5.0%). Post‐thaw semen quality was assessed. These results showed that the extender containing 0.5% cinnamaldehyde exhibited significant protective effects (*p* < 0.05) on post‐thaw sperm plasma membrane and sperm viability compared to the control and other experimental groups. In conclusion, supplementing the extender with 0.5% cinnamaldehyde effectively preserved the quality of post‐thawed sperm from Kajli rams.

## Graphical abstract

This study evaluated the protective effects of cinnamaldehyde on Kajli ram sperm quality at 4°C and after freezing, using semen samples from 12 rams collected via an artificial vagina at 42°C. The semen was extended in a tris‐citric acid extender with varying cinnamaldehyde concentrations (0%, 0.5%, 1.0%, 2.5% and 5.0%), and post‐thaw quality was assessed. Results indicated that 0.5% cinnamaldehyde significantly improved sperm plasma membrane integrity and viability (*p* < 0.05), effectively preserving post‐thaw sperm quality.

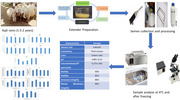

## 1 Introduction

Cryopreservation of sperm is a delicate process that requires precise control of the freezing and thawing rates. In addition, sperm cells are very fragile and cannot easily withstand the extreme conditions involved in cryopreservation. This fragility of sperm to cryopreservation varies not only between ejaculates but within ejaculates as well (Thurston and Watson 2002). As a result, fertility is reduced due to cryopreservation of semen (Lessard et al. 2000). Various factors contribute to the reduction in fertility, with the production of reactive oxygen species (ROS) being the most detrimental, causing significant damage to the sperm. ROS are essential for sperm capacitation, various signalling mechanisms and the reactivity of the acrosome (Gürler et al. 2016). However, oxidative stress is created when ROS production exceeds the required amount during cryopreservation. ROS initiates a series of reactions with membrane polyunsaturated fatty acids (PUFAs), collectively known as lipid peroxidation (LPO) that not only hinders physiology but also suppresses the survival rate of sperm (Len et al. 2019). Although the sperm has its own antioxidant defence system, it is not sufficient to deal with the ROS generated during cryopreservation. To manage this oxidative stress resulting from excessive ROS, semen extenders are often supplemented with exogenous antioxidants (Liu et al. 2021). These exogenous antioxidants safeguard sperm integrity from the harmful effects of potentially toxic‐free radicals generated during cryopreservation (Banday et al. 2017). The composition of the ram sperm membrane makes it even more vulnerable to ROS. The sperm plasma membrane has a high content of lipids and PUFAs, which make it susceptible to LPO and result in the production of peroxides, superoxide and hydroxyl radicals (Ferrusola et al. 2009). These highly reactive species then attack the plasma membrane, make it more fragile and rupture it, thereby severely affecting and damaging sperm motility and viability.

Cinnamaldehyde (CA) is the main ingredient of cinnamon, obtained from the stem bark of *Cinnamomum cassia*. This chemical exhibits various biological properties, such as antibacterial, anti‐inflammatory and anticancer activities (Suryanti et al. 2018). Literature has reported that CA has been used as a ROS scavenger, thus depicting its antioxidant properties (Davaatseren et al. 2017). It was found that CA helps protect against oxidative stress, as seen by lower levels of the oxidation damage marker malondialdehyde (MDA) in the blood of high‐fat diet‐fed mice after CA treatment (Li et al. 2019). Another study by Nour et al. (2018) confirmed this, showing that giving CA to rabbits on a high‐cholesterol diet for 4 weeks reduced atherosclerosis. CA treatment also decreased MDA and myeloperoxidase (MPO) levels while increasing antioxidant enzymes superoxide dismutase (SOD) and catalase (CAT) in aortic tissues (Nour et al. 2018). In addition, CA can balance ROS and MDA levels in ox‐LDL‐stimulated endothelial cells (ECs) by increasing SOD levels through the activation of Nrf2, a key factor for antioxidant gene expression, as reported by Li and colleagues (Nour et al. 2018). Supporting this, other lab experiments showed that CA boosts the p38 pathway and activates Nrf2, leading to the production of the antioxidant enzyme hemeoxygenase‐1 (HO‐1) in ECs exposed to hydrogen peroxide (Kim et al. 2020).

In the case of sperm cryopreservation, the cinnamon extract has been used in goats (Ariyan et al. 2021) and red deer (Sánchez‐Rubio et al. 2018) where it led to significant improvement in sperm quality parameters such as motility, viability, acrosome status and decline in MDA concentration and suppressed ROS generation as well. However, the use of CA in ram sperm cryopreservation has not been reported so far. Therefore, the objective of this study was to evaluate the effect of CA on Kajli ram sperm quality. It has been hypothesized that supplementing the CA in tris‐egg yolk‐glycerol (TEYG) extender may enhance the quality of equilibrated and post‐thaw Kajli ram sperm. The protective effects were evaluated by assessing sperm motility and viability and the integrity of the sperm membrane, sperm acrosome and DNA.

## 2 Materials and Methods

### 2.1 Management and Semen Collection

Twelve Kajli rams (1.5–2 years) were kept and managed at the Livestock Experimentation Station (LES), Khushab. Uniform feeding and management environment was provided to the animals during the course of the study. The rooms housing the animals were designed in such a way that they ensured proper ventilation of air during summer and adequate heat supply during the winter season. The breeding rams were fed seasonal fodder such as *Trifolium alexandrium* (berseem), *Medicago sativa* (alfalfa) and other seasonal crops. Apart from that, the animals were also fed with good quality concentrate including bran (chokar) and cotton seed cake (khal banola). Clean drinking water was in easy access and provided as per the ad libitum. Proper showers were installed in the farm where animals were showered to keep them neat and clean. Moreover, sheep shearing was done at regular intervals for the same purpose. The animals were also properly vaccinated.

### 2.2 Extender Preparation

TEYG was used as a freezing medium. The ingredients of the extender involved 12.112 g (hydroxymethyl) aminomethane (Sigma‐Aldrich, USA), 6.7 g citric acid and 5 g fructose (Sigma‐Aldrich, USA). All these ingredients were then dissolved in 365 mL of distilled water on a hotplate at 65°C. The mixing was followed by the addition of 100 µg/mL streptomycin (Sigma‐Aldrich, USA) and 50 mL of egg yolk. The temperature was then lowered to 35°C and glycerol was added gradually (Ariyan et al. 2021). The extender was divided into five experimental groups to which different concentrations (0.5%, 1.0%, 2.5%, 5.0% and 0%) of CA (UniChem, USA) were added.

### 2.3 Semen Collection

For each experiment, two consecutive ejaculates were collected using an artificial vagina (42°C) from 12 Kajli rams during the peak breeding season in February and March, in 2023 at weekly intervals for a period of 3 weeks (replicate). Semen from each ram (two ejaculates/ram/week) was immediately transferred to the laboratory. For further analysis, samples with an average volume around 0.90 mL, greater than 70% motility, thick milky white appearance, pH 6.8–7.1 with vigorous and dense moving waves, and a consistency between 70% and 85% were selected (Qadeer et al. 2024).

### 2.4 Semen Processing

Semen samples are frozen with cryopreservation medium or TEYG freezing medium. The samples are diluted slowly, in one step, with the TEYG freezing medium so that the final sperm concentration is 400 × 10^6^ sperm/mL and cooled to 5°C over 90–120 min. The samples are loaded into 0.5 mL straws and frozen. Samples are frozen by using box freezing: Samples are placed on a rack and frozen in liquid nitrogen vapour (4 cm above liquid nitrogen) for 10 min and plunged into the liquid nitrogen for storage. Thawing was done after 24 h to evaluate the effect of CA on post‐thaw quality.

### 2.5 Post‐Thaw Analysis

#### 2.5.1 Sperm Progressive Motility

Sperm motility was measured using a camera‐fitted compound microscope. A drop of sample from each of the groups was placed on a labelled slide and observed under a camera‐fitted microscope (SY‐B125, China). The same procedure was repeated thrice and the mean was taken (Shahat et al. 2021).

#### 2.5.2 Plasma Membrane Integrity

Hypo‐osmotic swelling test (HOST) was used to evaluate the integrity of the plasma membrane (Fukui et al. 2004). 0.73 g sodium citrate (Sigma‐Aldrich, USA) and 1.35 g fructose (Sigma‐Aldrich, USA) were mixed in distilled water for the preparation of the HOST solution. 50 µL of the thawed sample was then mixed with 500 µL of HOST solution in a test tube and incubated for 30–40 min at 37°C. About 50 µL of this mixture was then placed on a pre‐warmed slide and observed under camera‐fitted microscope (SY‐B125, China) under 40X magnification (Qadeer et al. 2014). A total of 7200 sperm were examined per treatment (200 per each of the three replicates for each of the 12 rams). Swollen tails indicated intact, biochemically active sperm membranes, whereas unswollen tails indicated disrupted, inactive sperm membranes.

#### 2.5.3 Sperm Viability

Eosin‐nigrosin method was used for measuring sperm viability. Eosin can cross a damaged membrane and give it a red colour. Nigrosin provides a dark background to make visualization of live unstained cells easy. Separate solutions of both stains were prepared by mixing 1 g of each in 100 mL of distilled water on a hotplate. A drop of eosin solution and a relatively bigger drop of nigrosin solution (Analar BDH Laboratory Supplies, England) were mixed with a drop of thawed semen sample on a pre‐warmed slide. A smear was prepared, air‐dried, and a total of 200 sperm were evaluated in each smear under camera‐fitted compound microscope (SY‐B125, China) at 40X magnification. Live sperm were indicated by unstained or white sperm heads while pink heads were an indication of dead sperms (Agarwal et al. 2016).

#### 2.5.4 Acrosome Integrity

Giemsa staining method was used for assessing the integrity of the acrosome. The staining solution was prepared by mixing 2 mL phosphate buffered saline (PBS) (Sigma‐Aldrich, USA) and 3 mL absolute Giemsa (Sigma‐Aldrich, USA) in 35 mL distilled water on a hotplate. About 50 µL of thawed semen was put on a pre‐warmed slide and a smear was prepared. The smear was then fixed using methanol for 10 min. The slide was then rinsed and placed in a staining jar for 3 h. A total of 7200 sperm were examined per treatment (200 per each of the three replicates for each of the 12 rams). A purple head is an indication of an intact acrosome while a damaged acrosome is indicated by a pale lavender head (Prihantoko et al. 2020).

#### 2.5.5 DNA Damage

DNA damage was measured using an acridine orange stain. Evaluation of DNA damage also involved Carnoy's solution which was prepared using glacial acetic acid (Sigma‐Aldrich, USA) and methanol (Sigma‐Aldrich, USA) in a ratio of 1:3, respectively. A drop of thawed sample was put on a slide and the air‐dried slide was then placed for 2 h in Carnoy's Solution. The slides were taken out and incubated at 75°C in a water bath for 5 min. The slides were then stained with acridine orange solution for 10 min in the dark. The slides were washed, air‐dried and examined under a fluorescent microscope (BS‐2070FT, Bestscope China) at a magnification of 100X. A total of 7200 sperm were examined per treatment (200 per each of the three replicates for each of the 12 rams). The green colour was an indication of sperms with intact double strands while damaged DNA emitted orange/red fluorescence (Varghese et al. 2009).

### 2.6 Statistical Analysis

The effect of supplementation of CA in extender on the quality of Kajli ram sperm was analysed by analysis of variance using SPSS‐20. Specific differences were identified with Tukey's test. All statistical analyses were performed at *α* =  0.05.

## 3 Results

**FIGURE 1:**
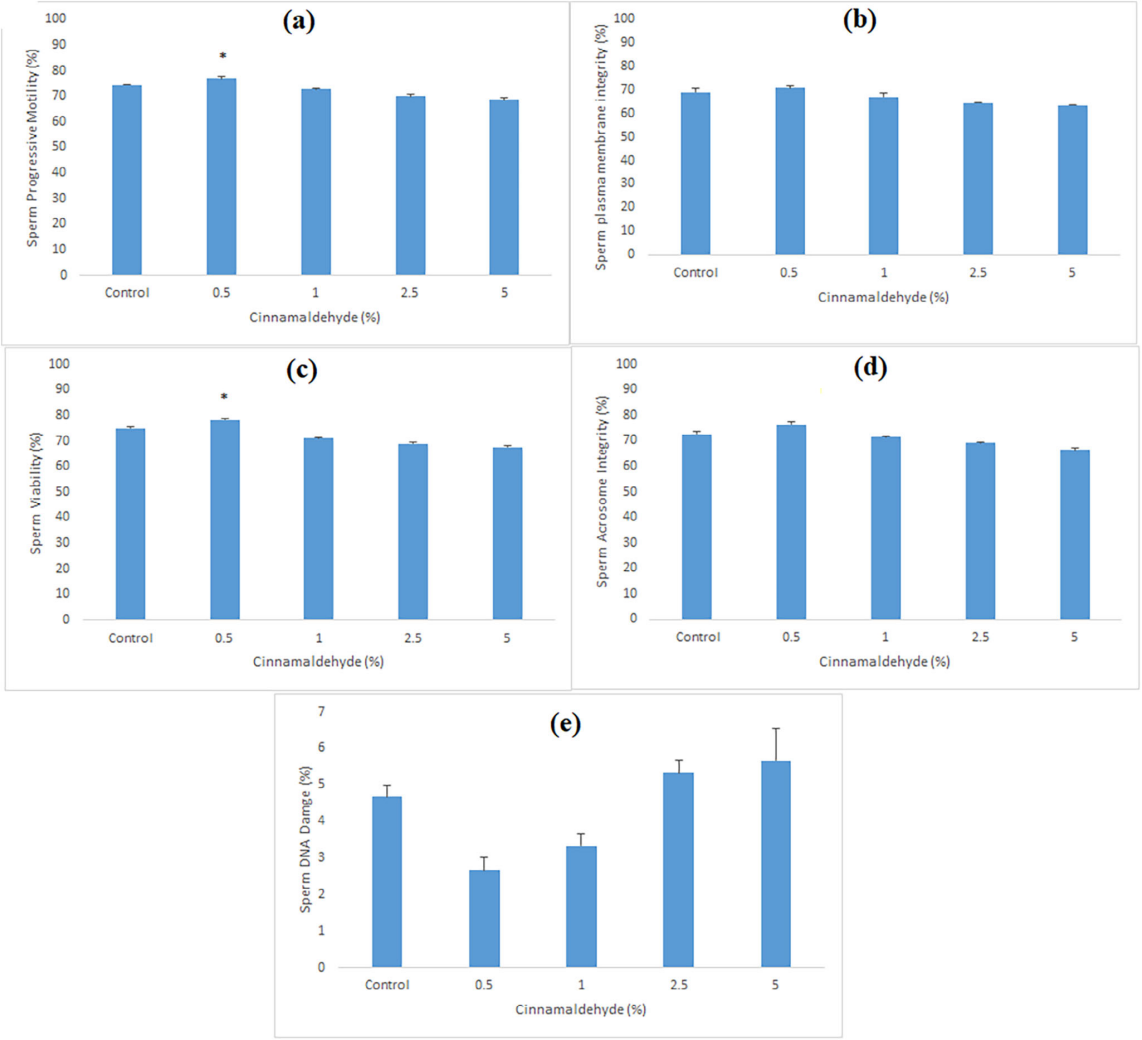
Effect of cinnamaldehyde supplementation (0%, 0.5%, 1.0%, 2.5% and 5.0%) in the extender at 4°C on sperm progressive motility (a), sperm plasma membrane integrity (b), sperm viability (c), sperm acrosome integrity (d) and sperm DNA damage (e) in Kajli ram sperm. Bars marked with an asterisk sign indicate significant differences (*p* < 0.05) from the control group.

**FIGURE 2:**
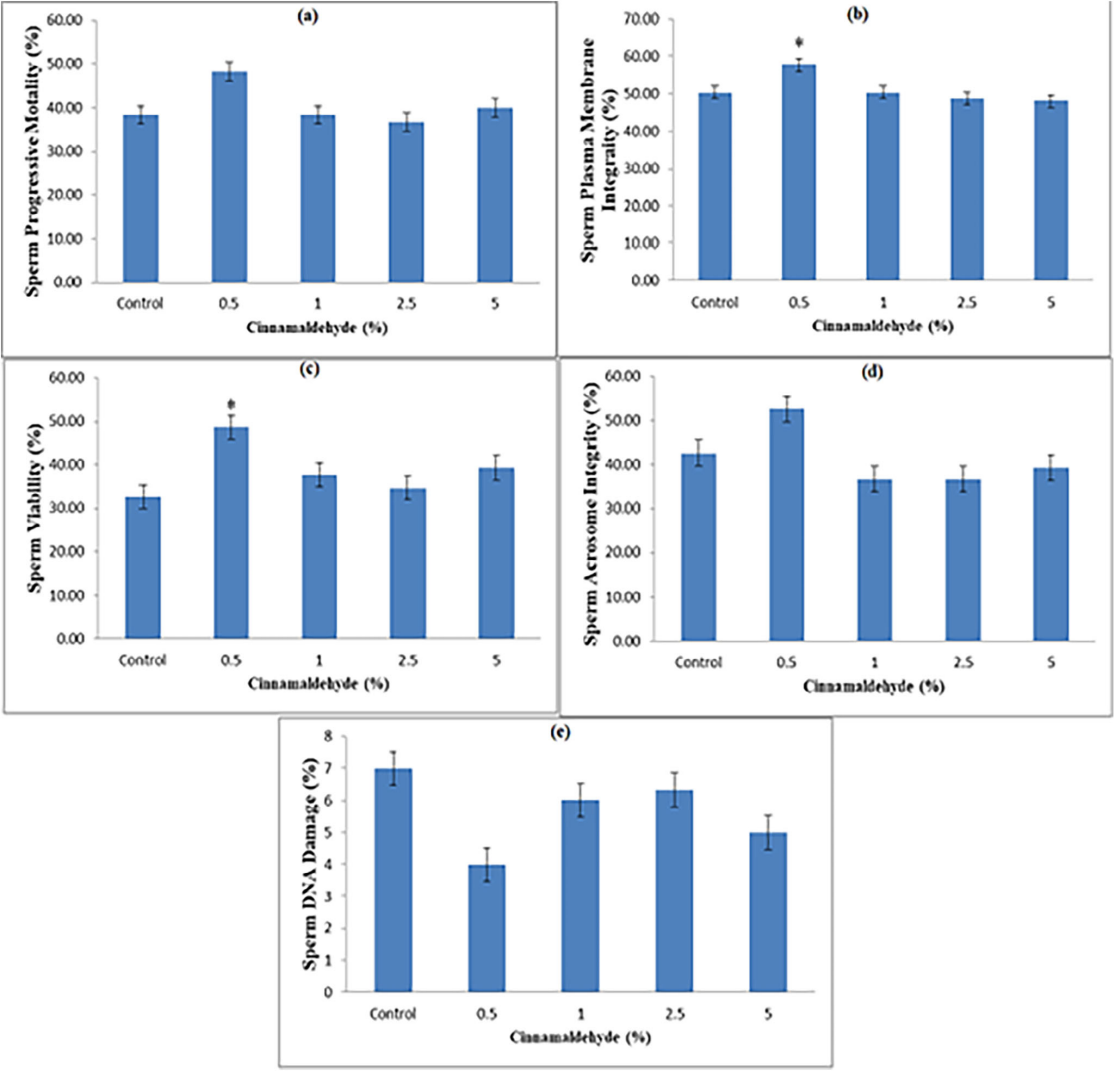
Effect of cinnamaldehyde supplementation (0%, 0.5%, 1.0%, 2.5% and 5.0%) in the extender on post‐thaw sperm progressive motility (a), sperm plasma membrane integrity (b), sperm viability (c), sperm acrosome integrity (d) and sperm DNA damage (e) in Kajli ram sperm. Bars marked with an asterisk sign indicate significant differences (*p* < 0.05) from the control group.

Only qualified semen samples meeting standard criteria of volume, concentration and motility were selected for further evaluation (qualifying criteria are presented in Table 1). Results regarding sperm progressive motility, sperm plasma membrane integrity, sperm viability, acrosome integrity and DNA damage at 4°C and after freezing are presented in Figures 1 and 2. Sperm motility at 4°C and after post‐thaw is shown in Figures 1a and 2a, respectively. The experimental group with 0.5% CA at a 7.5 µL/mL concentration at 4°C reported better results (significant; *p* < 0.05) compared to the control; however, after freezing this concentration showed better results (non‐significant; *p* > 0.05). Results regarding the effect of CA on membrane integrity at 4°C and after post‐thaw is shown in Figures 1b and 2b, respectively. The extender with 0.5% CA significantly (*p* < 0.05) protected the sperm plasma membrane integrity compared to the control after post‐thaw evaluation. However, at 4°C non‐significant difference was found. The effects of different concentrations of CA on sperm viability are shown in Figures 1c and 2c. Significantly improved sperm viability (*p* < 0.05) was observed in the extender with 0.5% CA both at 4°C and after post‐thaw. The effects of different concentrations of CA on acrosome integrity and sperm DNA damage are represented in Figures 1d,e and 2d,e. These results show non‐significant (*p* > 0.05) differences between the control and experimental groups both at 4°C and after post‐thaw.

**TABLE 1:** The quality parameters of fresh semen samples.

Characteristics	Average
Volume (mL)	0.85 ± 20
Consistency	Thick viscous
Colour	Cream colour
pH	6.88 ± 0.22
Concentration (× 10^6^ sperm/mL)	4.273 ± 401
Motility	90%
Viability	85%
Acrosome integrity	88%
Plasma membrane integrity	86%

## 4 Discussion

The sperm comes across a variety of stresses during cryopreservation. These stressors result in a significant decline in sperm quality. ROS are one of these stresses that are a major threat to sperm viability. Although the sperm has its own antioxidant defence mechanism but it is not sufficient to counter ROS generated during cryopreservation. Various chemicals have been used in the semen extenders for their antioxidative properties. This study involved the supplementation of CA as an antioxidant to protect sperms against oxidative stress. Sperm motility serves as the key to successful fertilization. The equilibration process involves the addition of cryoprotectants and antioxidants, which creates an osmotic stress for the sperm and also leads to the generation of ROS. The abundance of PUFAs in the ram sperm plasma membrane makes it more vulnerable to ROS, causing their oxidation and damaging the membrane integrity. As a result, membrane integrity gets affected, which in turn suppresses the motility of sperm (Sundararaman and Edwin 2008).

In the present study, a 0.5% concentration of CA was found to be optimum for sperm motility at 4°C and after freezing. Sperm motility then started to decline with the increase in concentration of CA. The lowest motility parameter was shown by 5.0% CA in the extender. This decrease in motility with increasing concentrations can be attributed to increasing viscosity which limits the movement and also exerts toxic effects. Higher concentrations of antioxidants are also reported to affect the mitochondria, affecting their energy production abilities. This eventually leads to a decline in motility (Shi et al. 2020).

Both equilibration and thawing processes involve various stressors that are antagonistic to sperm viability and bring about a considerable decline in viability. The major driving force behind this decline is the generation of ROS which damages the vital organelles of the sperm, thereby leading to the death of the sperm (Galantino‐Homer et al. 2006). Viability was also reported to be highest in the extender with 0.5% CA both at 4°C and after freezing. Higher concentrations proved to be detrimental to sperm viability. This can be attributed to the osmotic dehydration of sperm. Higher concentrations of cryoprotectants and antioxidants make the outside environment hypertonic, thereby drawing water out of the sperm. This change in solute equilibrium causes shrinkage of the sperm, ultimately leading to its rupture and death. Opposite is the case during the thawing process where solute imbalance causes water to move inside, thereby causing it to swell up and rupture.

The vulnerability of the ram sperm to ROS emerges from PUFAs, which are present in abundance in the ram sperm plasma membrane, thereby making it more susceptible to ROS attack and affecting membrane integrity. Plasma membrane integrity was reported to be significantly high at 0.5% CA as compared to the rest of the experimental and control groups as well. High membrane integrity at lower concentrations may be attributed to the antibacterial and antioxidative properties of CA (Carrera‐Chávez et al. 2020). The decline in membrane integrity may be due to the cytotoxic effects of higher concentrations of CA. These results are in agreement with the study evaluating the antioxidative properties of *Moringa oleifera* on ram sperm where a lower concentration of the plant was found to be more protective for the membrane integrity (Carrera‐Chávez et al. 2020). The higher concentrations are reported to disturb the lipid and protein content of the plasma membrane, which are known to maintain the fluid nature of the membrane. The alterations in the membrane's fluidity in turn affect its integrity.

Acrosome integrity was also found to be highest in the extender with 0.5% CA. The mechanism of action through which CA protects acrosome integrity remains unclear but studies have also associated acrosome integrity with that of the plasma membrane. A higher percentage of plasma membrane integrity ensures the same result for acrosome integrity and vice versa. This is because antioxidants and cryoprotectants are more effective at an optimum concentration and their effects deteriorate above or below that concentration. The antioxidative properties of CA help suppress oxidative stress to preserve the integrity of acrosome.

DNA damage is reported to be the main reason behind infertile males. ROS formed during cryopreservation may damage the DNA as well. The endogenous antioxidants are not sufficient to protect the DNA from ROS. Exogenous antioxidants are supplied to compensate for this insufficient antioxidant status and boost the sperms defence against oxidative damage (Zini et al. 2009). Though the lowest DNA damage was observed in the extender with 0.5% CA, it was not significantly different from the other experimental and control groups. CA upregulates the expression of various antioxidant enzymes such as CAT and SOD, which in turn enhance the antioxidant profile of the sperm. It also reduces the MDA level (Mousa et al. 2021).

The protective effects of CA on post‐thaw sperm quality observed in this study are consistent with its well‐documented antioxidant properties in various biological systems. However, the present study did not directly measure antioxidant enzyme activities such as CAT, SOD and glutathione peroxidase (GPx) in semen samples. These enzymes play a critical role in protecting sperm cells from oxidative stress, particularly during cryopreservation. Future studies are warranted to explore the precise biochemical mechanisms underlying CA's action, including comprehensive antioxidant profiling in ram spermatozoa. Such investigations would further clarify whether the observed improvements in sperm quality are directly linked to modulation of antioxidant defence systems.

## 5 Conclusions

This study demonstrated that incorporating 0.5% CA into the TEYG extender provided substantial protection for Kajli ram sperm. Specifically, it significantly improved sperm plasma membrane integrity and sperm viability. Further studies are warranted to test the benefits of this protocol on conception success in livestock breeding of Kajli ram and related species.

## Author Contributions

**Saima Qadeer**: conceptualization, funding acquisition, methodology, project administration, resources, supervision, writing – original draft. **Asma Ashraf**: investigation, methodology. **Naila Perveen**: conceptualization, data curation, formal analysis, methodology, writing – original draft. **Muhammad Asad**: formal analysis, software. **Asma Ul Husna**: validation, writing – review and editing. **Asima Azam**: validation, writing – review and editing. **Muhammad Umer Farooq**: data curation, software. **Abdullah S. Alhomida**: funding acquisition, writing – review and editing. **Haseeb A. Khan**: funding acquisition, writing – review and editing.

## Ethics Statement

This study was approved by the ethical committee of the University of Education, Lahore, for the use of animals.

## Conflicts of Interest

The authors declare no conflicts of interest.

## Peer Review

The peer review history for this article is available at https://www.webofscience.com/api/gateway/wos/peer‐review/10.1002/vms3.70484.

## Data Availability

The data that support the findings of this study are available from the corresponding author upon reasonable request.
